# Endometritis decreases the population of uterine neurons in the paracervical ganglion and changes the expression of sympathetic neurotransmitters in sexually mature gilts

**DOI:** 10.1186/s12917-021-02949-z

**Published:** 2021-07-10

**Authors:** Bartosz Miciński, Barbara Jana, Jarosław Całka

**Affiliations:** 1 Department of Clinical Physiology, Faculty of Veterinary Medicine, University of Warmia and Mazury, Oczapowskiego 13, 10-719 Olsztyn, Poland; 2 Division of Reproductive Biology, Institute of Animal Reproduction and Food Research, Polish Academy of Sciences, Tuwima 10, 10-748 Olsztyn, Poland

**Keywords:** Autonomic nervous system, Chemical coding, Pig, Endometritis, Immunocytochemistry

## Abstract

**Background:**

The focus of the study was to examine the impact of the inflamed uterus on the population of the paracervical ganglion (PCG) uterus-innervating perikarya and their chemical coding. Fast Blue retrograde tracer was injected into the wall of uterine horns on the 17th day of the first studied estrous cycle. After 28 days, either *Escherichia coli* suspension or saline was applied to the horns of the uterus, whereas the control group received laparotomy only. Eight days after the above-mentioned procedures, uterine cervices with PCG were collected. Both macroscopic and histopathologic examinations confirmed severe acute endometritis in the *Escherichia coli*-injected uteri. The double immunofluorescence method was used to analyze changes in the PCG populations coded with dopamine-β‐hydroxylase (DβH) and/or neuropeptide Y (NPY), somatostatin (SOM), vasoactive intestinal polypeptide (VIP) and neuronal isoform of nitric oxide synthase (nNOS).

**Results:**

The use of *Escherichia coli* lowered the total number of Fast Blue-positive neurons. Moreover, an increase in DβH+/VIP+, DβH+/NPY+, DβH+/SOM + and DβH+/nNOS + expressing perikarya was noted. A rise in non-noradrenergic VIP-, SOM- and nNOS-immunopositive populations was also recorded, as well as a drop in DβH-positive neurotransmitter-negative neurons.

**Conclusions:**

To sum up, inflammation of the uterus has an impact on the neurochemical properties of the uterine perikarya in PCG, possibly affecting the functions of the organ.

## Background

One of the most common pathologic conditions in domestic animals generating severe economic problems for breeders, such as increased medical costs and deteriorated reproductive indicators, is uterus inflammation. This disease develops in response to non-infectious agents and, more importantly, to bacteriological factors. Mainly occurring after parturition, metritis/endometritis might also be evoked through natural mating or insemination in primiparous animals [1–3]. Its mechanism is based on the problems with uterine involution, often matched with immunological reaction, which consists of an increase in the levels of proinflammatory cytokines and other mediators, including tumor necrosis factor-α, interleukins - IL-1β, IL-6, IL-8, nitric oxide (NO), prostaglandins – PGF2α, PGE2, as well as leukotrienes - LTB4 and LTC4 [4–6].

The paracervical ganglion (PCG; Frankenhauser’s ganglion) is an unprecedented formation belonging to the autonomic nervous system. It contains both sympathetic (noradrenergic) as well as a parasympathetic (cholinergic) component and is a part of a larger pelvic plexus innervating organs of the urinary tract and reproductive system [7–9]. The porcine uterus is supplied by nerve terminals from many autonomic and sensory ganglia including the PCG. This has been confirmed in the past with the use of Fast Blue (FB) fluorescent retrograde neuronal tracer, which indicated the existence of uterus-innervating neurons inside the structures of Frankenhauser’s ganglion [10]. Studies focusing on the double immunohistochemical staining of PCG perikarya in rats, cats and guinea pigs acknowledged the expression of vesicular acetylcholine transporter (VAChT) and choline acetyltransferase (ChAT) as markers of cholinergic neurons as well as noradrenaline (NA), tyrosine hydroxylase (TH) and dopamine beta hydroxylase (DβH) as markers of the noradrenergic nerve cell population [11, 12]. The coexistence of various substances in these types of neurons, including vasoactive intestinal polypeptide (VIP), neuropeptide Y (NPY), galanin (GAL), neuronal isoform of NO synthase (nNOS), somatostatin (SOM), galanin (GAL) and substance P (SP) has been confirmed by other authors [9, 13–18]. Literature on the chemical coding of uterine-innervating populations of nervous cells in the porcine PCG is insufficient [9, 10]. Moreover, there is a total lack of information regarding the impact of metritis/endometritis on the expression of any of these substances in either the noradrenergic or cholinergic neurons in PCG.

Since pigs are embryologically, anatomically and physiologically similar to human beings, their significance in any type of biomedical research, including studies on the reproductive system, is invaluable [19, 20]. The authors intended to acquire data on the pig model which may help both animals and breeders by improving breeding indicators, survivability and profitability. In a broader aspect, the study may be valuable to women suffering from uterus inflammation, as it is hoped that these results will serve as a basis for the development of novel therapeutic agents, such as neurotransmitter analogues that can be administered to humans.

Literature describing the morphology and chemical coding of the uterine-perikarya in relation to the inflammation of the uterus is lacking. The available data show that endometritis in rats caused changed behavior as a probable response to visceral pain, as well as an augmented population of SP-immunoreactive neurons in the sensory ganglia (dorsal root ganglia; DRGs) [21, 22]. Three of the authors’ published studies reported that, in gilts, endometritis has an impact on the uterus-supplying neuronal populations. It was first reported that in *Escherichia coli* (E. coli)-evoked inflammation, the number of nerve fibers diminished, including noradrenergic fibers [23]. A subsequent article on the effect of uterine inflammation on sensory ganglia presented results showing decreasing numbers of perikarya in DRGs [24], whereas the most recent article described a drop in the total number of uterine supplying neurons in caudal mesenteric ganglion (CaMG) in response to the same disturbance [25]. Based on these results it may be hypothesized that metritis/endometritis affects the neurochemical properties of uterus-innervating neuronal cells in PCG in sexually mature gilts. Understanding the morphological and neurochemical changes of PCG uterine neurons in response to uterine inflammation may be important for the course and consequences of a pathological process. Therefore, the aim of the current study was to test this hypothesis by an examination of (1) the total number of uterine perikarya, as well as (2) determination of the number of cells expressing DβH and/or VIP, NPY, SOM and nNOS in this ganglion.

## Results

### The number and distribution of uterine perikarya in the PCG

The most numerous concentrations of uterus-innervating PCG-labeled neurons were identified in the zone of the first 5 centimeters behind the portio vaginalis cervicis, gradually diminishing in the cranial direction. The total number of FB-positive neurons in the E. coli group was lower (*p* < 0.001) than in the control and saline groups (351 ± 9.20 vs. 588 ± 16.70, 610 ± 13.24, respectively). It is worth noting that in all groups left-side paracervical ganglia always had a lower (*p* < 0.001) amount of uterus-innervating perikarya than the right-side ganglia. Moreover, the decrease in the number of left-side neurons after bacterial administration was more significant vs. the control (*p* < 0.001) group than the saline (*p* < 0.01) group. Additionally, the right-side ganglia of the saline group presented a statistically more significant decrease in the number of the FB-positive neurons than the left-side formations of the same group in relation to the bacteria-treated population (right-side: *p* < 0.001 vs. left-side: *p* < 0.01).

**Fig. 1:**
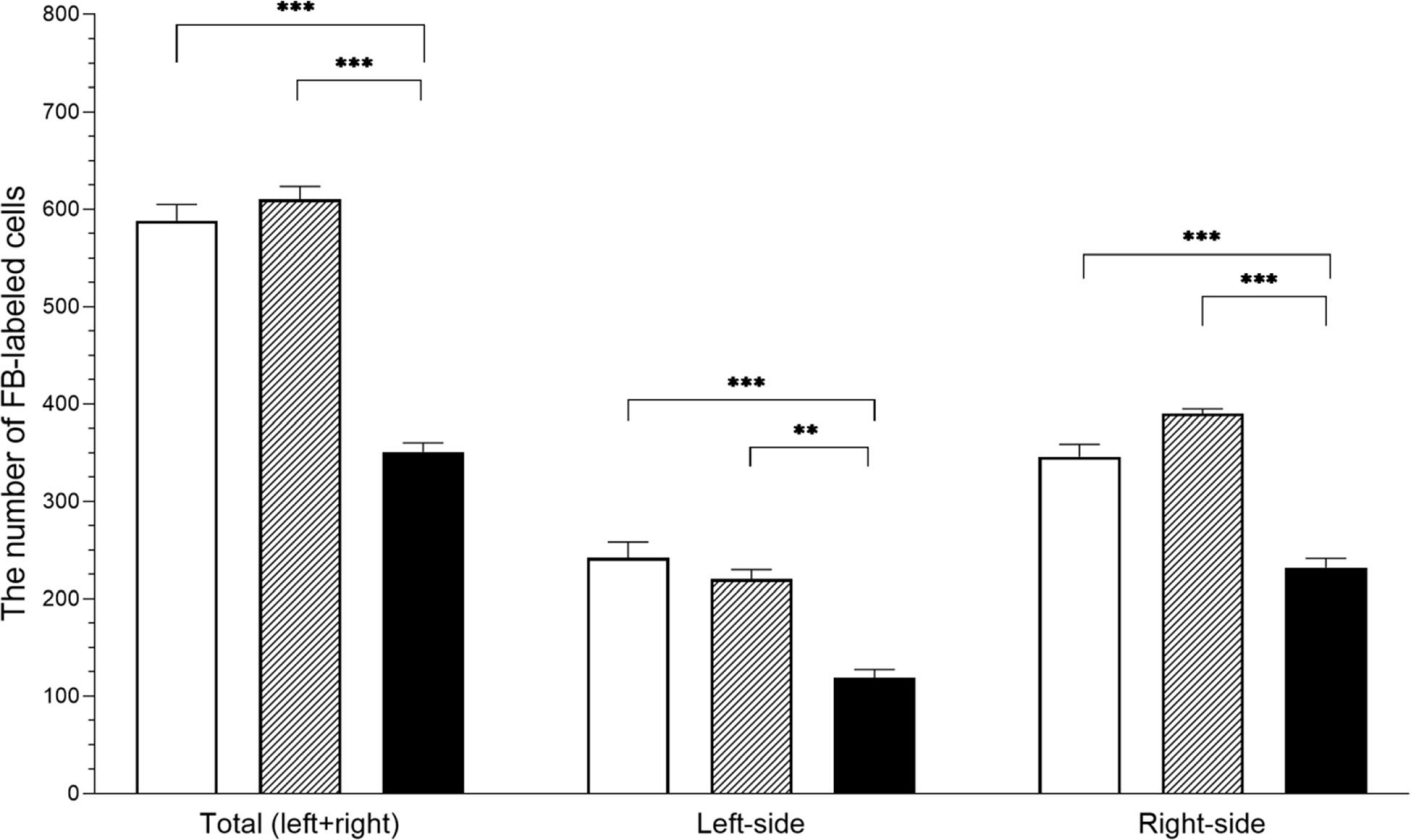
Total, left-side and right-side perikaryal cell count (mean ± SEM) in the paracervical ganglion (PCG) projecting to the uterus of gilts from the control (white bars), saline (grey bars) and E. coli (black bars) groups (** *p* < 0.01 and *** *p* < 0.001 show the differences between all groups in the same ganglion region)

The total number of perikarya, as well as the distinction into left and right-side populations of the PCG perikarya, is depicted in Fig. 1.

### The number of uterine-supplying neurons containing DBH, SOM, VIP, NPY and nNOS in the PCG

**Fig. 2:**
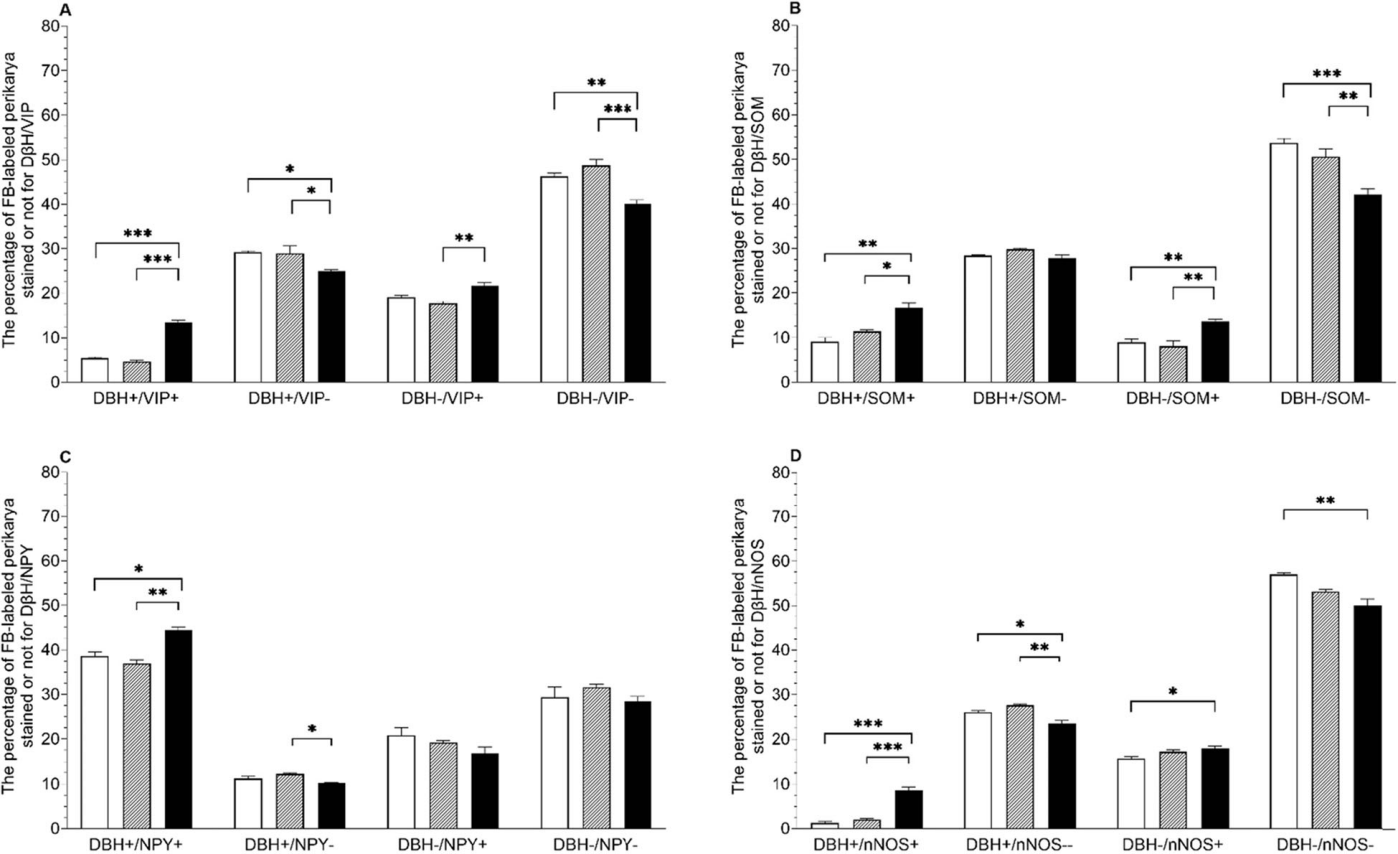
The populations (expressed as percentages, mean ± SEM) of uterine perikarya expressing dopamine-β-hydroxylase (DβH) and/or vasoactive intestinal polypeptide (VIP) (**A**), DβH and/or somatostatin (SOM) (**B**), DβH and/or neuropeptide Y (NPY) (**C**) and DβH and/or neuronal isoform of nitric oxide synthase (nNOS) (**D**), as well as those without these substances in the PCG of gilts from the control (white bars), saline (grey bars) and E. coli (black bars) groups. Data are expressed as percentages of the total population of uterine perikarya stained for two substances in each group, accepted as 100 %. * *p* < 0.05, ** *p* < 0.01 and *** *p* < 0.001 show differences between all groups for the same population of uterine perikaryal

**Fig. 3:**
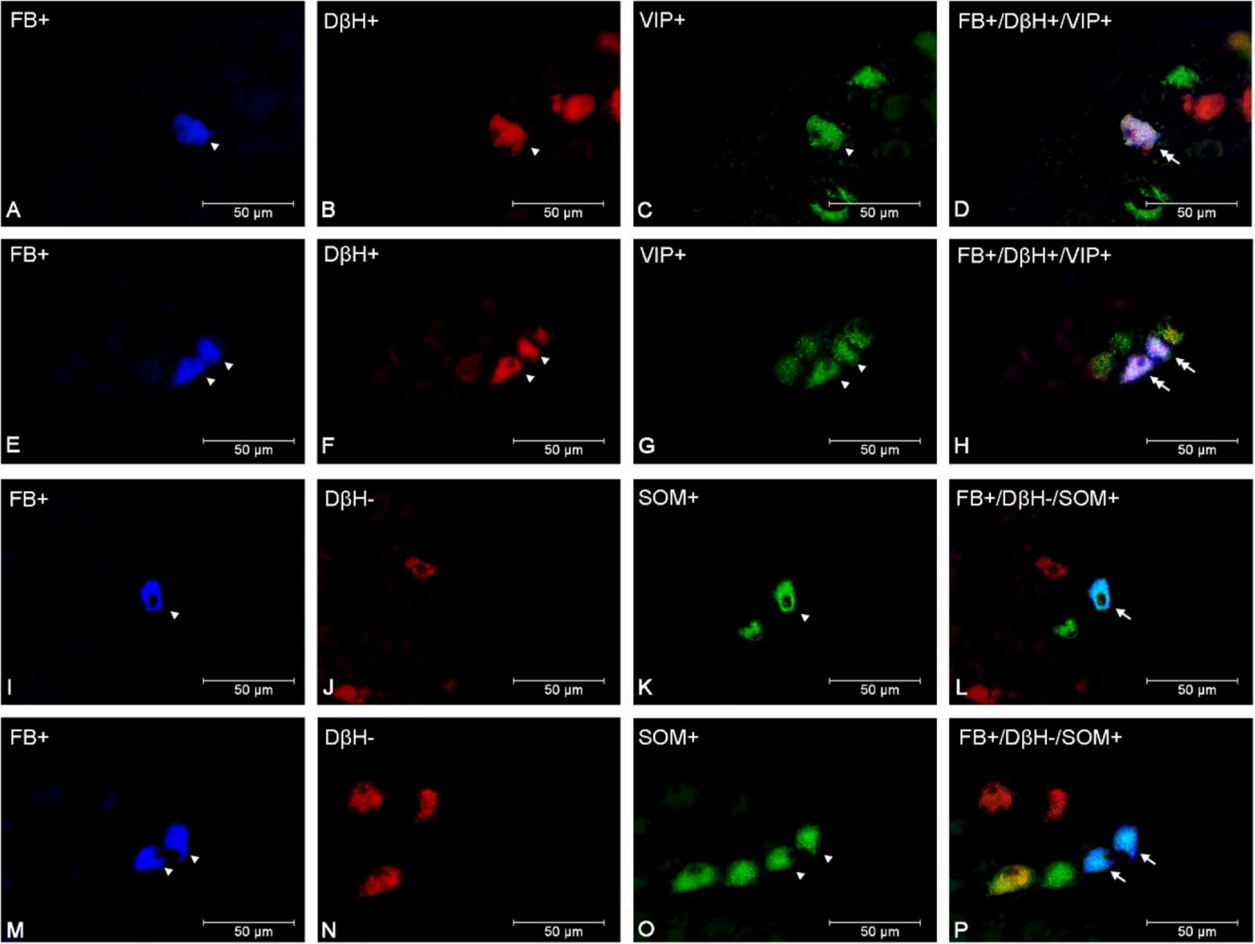
Micrographs demonstrating the presence of DβH (**B**, **F**, **J**, **N**), VIP (**C**, **G**) and SOM (**K**, **O**) in the PCG uterine perikarya of gilts from the control (**A-D**), saline (**I**-**L**) and E. coli (**E**-**H**, **M**-**P**) groups. The arrowhead indicates a Fast Blue (FB)-positive neuron, a perikaryon immunoreactive to DβH and VIP and a perikaryon immunoreactive to SOM. The double arrow indicates an FB-positive uterine neuron expressing DβH and VIP. The arrow indicates an FB-positive perikaryon expressing SOM. The photographs (**D**, **H**, **L**, **P**) were made by digital superimposition of three color channels: FB-positive (blue), DβH-positive (red) and SOM- or VIP-positive (green). One DβH and VIP immunoreactive uterine neuron is visible in the gilt of the control group (**A**-**D**). In the PCG of the E. coli group, an elevated number of perikarya expressing these substances are visible (**E**-**H**). One perikaryon expressing SOM, but not DβH, is present in the ganglion of the saline group (**I**-**L**). In the E. coli group, two perikarya expressing SOM are observed in the PCG (**M**-**P**)

**Fig. 4:**
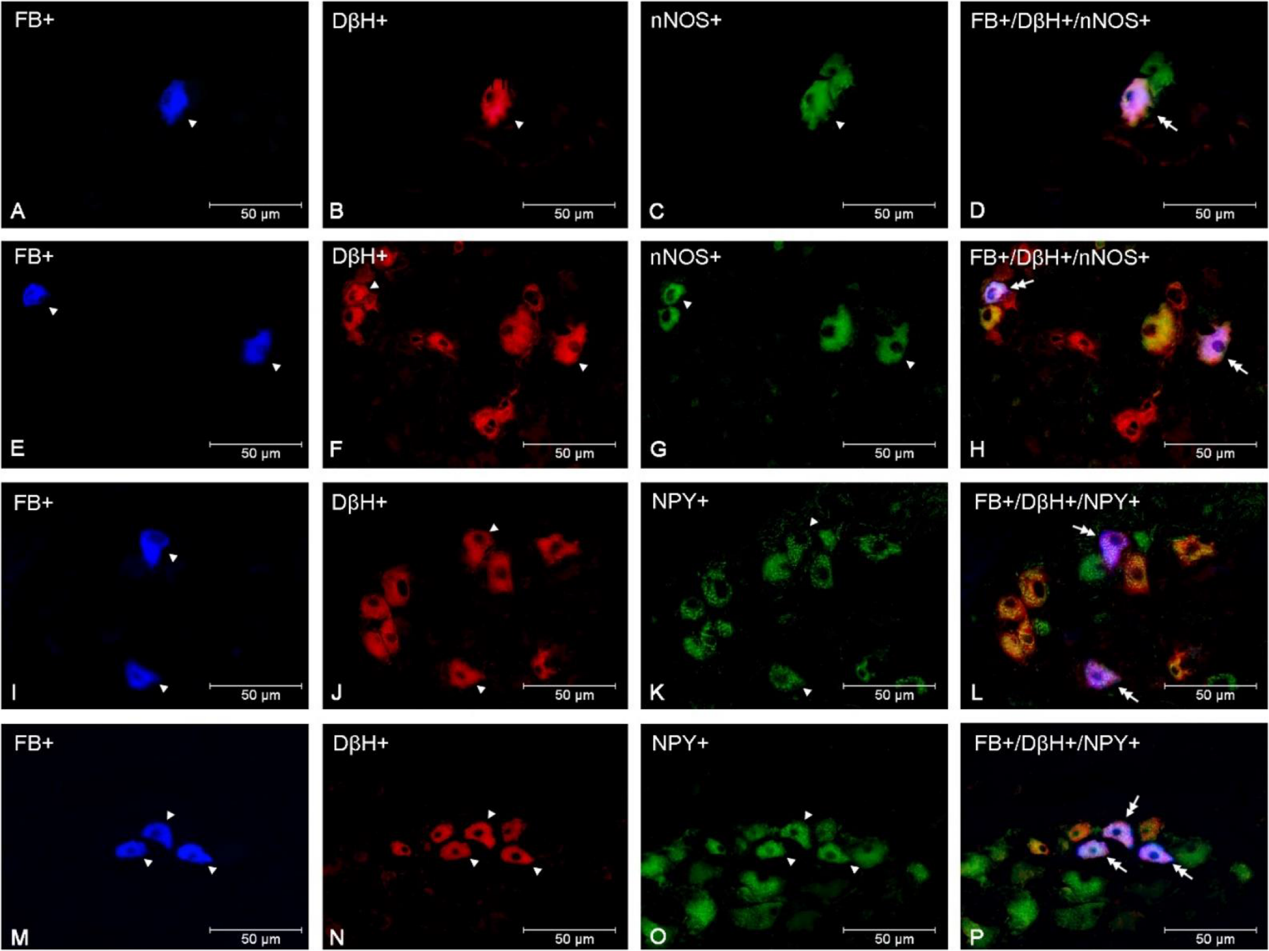
Micrographs demonstrating the presence of DβH (**B**, **F**, **J**, **N**), nNOS (**C**, **G**) and NPY (**K**, **O**) in the PCG uterine perikarya of gilts from the control (**A**-**D**), saline (**I**-**L**) and E. coli (**E**-**H**, **M**-**P**) groups. The arrowhead indicates a Fast Blue (FB)-positive neuron, a perikaryon immunoreactive to DβH and nNOS/NPY, a neuron immunoreactive to NPY, as well as an nNOS-immunoreactive perikaryon. The double arrow indicates an FB-positive uterine neuron expressing DβH and nNOS/NPY. The photographs were made by digital superimposition of three color channels: FB-positive (blue), DβH-positive (red) and nNOS- or NPY-positive (green). One perikaryon expressing nNOS and DβH is present in the ganglion of the control group (**A**-**D**). In the E. coli group, two perikarya expressing these substances are observed in the PCG (**E**-**H**). Two DβH and NPY immunoreactive uterine neurons are visible in the gilt of the saline group (**I**-**L**). In the PCG of the E. coli group, an elevated number of perikarya expressing these substances are visible (**M**-**P**)

In comparison to both control and saline groups, the number of DβH+/VIP + uterine perikarya statistically significantly increased in the PCG of the bacteria treated gilts (*p* < 0.001) (Figs. 2A and 3A-H), whereas the DβH-/VIP + population size was augmented (*p* < 0.01) in relation to the saline group (Fig. 2A). Moreover, the numbers of DβH+/VIP- in the E. coli group have noted a decrease (*p* < 0.05) when compared to both control and saline groups (Fig. 2A). The size of the DβH-/VIP- population in E.coli-injected gilts diminished as well in comparison to control (*p* < 0.01) and saline (*p* < 0.001) groups of animals (Fig. 2A). In the PCG of E. coli-injected gilts, the number of DβH+/SOM + neurons was higher than in the control (*p* < 0.01) and saline (*p* < 0.05) groups (Figs. 2B and 3I-P). Furthermore, a rise (*p* < 0.01) was noted in the bacterial group’ DβH-/SOM + coded neurons when compared to other groups, whereas a decrease was present in the DβH-/SOM- population in relation to both control (*p* < 0.001) and saline groups (*p* < 0.01) (Fig. 2B). E. coli treatment led to an increase in the population of the DβH+/NPY+ (Fig. 2 C, Fig. 4I-P) compared to other two groups (control group: *p* < 0.05, saline group: *p* < 0.01), although the DβH+/NPY- expressing population decreased (*p* < 0.05) its numbers in relation to the saline group (Fig. 2 C). Uterine inflammation also led to an increase in the number of DβH+/nNOS + neurons in comparison with two other examined groups (*p* < 0.001) (Figs. 2D and 4 A-H), a decrease in DβH+/nNOS- population (control group: *p* < 0.05, saline group: *p* < 0.01), a rise (*p* < 0.05) in the size of DβH-/nNOS + coded perikarya in relation to the control group, and evoked a decrease in the number of DβH-/nNOS- neurons (control group: *p* < 0.01) (Fig. 2D).

The numbers of uterine-supplying perikarya containing DβH and/or VIP, SOM, NPY, nNOS, as well as those lacking the expression of all these neurotransmitters in the PCG of the control, saline-and bacteria treated gilts are presented in Fig. 2.

## Discussion

This is the first study to demonstrate alterations in the number, as well as the chemical coding of the PCG uterus-supplying neurons in sexually mature gilts with the bacteria-induced inflammation of uterus. Inoculation of the E. coli suspension was performed in the early luteal phase of the estrous cycle when the increasing level of immunosuppressive progesterone is conducive to the development of inflammation. Moreover, this action resulted in an incidence of a severe form of acute inflammation since 17β-estradiol (E2), uterine PGF_2α_ and LTs levels (which are considered to have the immunostimulating effect) are very low during this phase [26, 27]. On the microscopic level, such a form is diagnosed when the number of neutrophils is highly increased and luminal epithelium and/or glands are damaged [1]. Such a state was histopathologically proven and the results of such an examination were presented in a previous study by the authors [24]. It is worth noting that saline inoculation did not have a statistically significant impact on the total number of uterine-perikarya or any of the neurons immunoreactive to substances examined in this manuscript. These numbers were fairly similar, which is presented in the results.

The number of uterine perikarya in total was significantly decreased. Such an occurrence has a negative impact on uterus physiology and may imply that, due to innervation degradation, its immunity to inflammation, as well as adaptation capabilities and regulatory potential are impaired. The overall number of uterine neurons in the left-side PCGs was found to be lower than in the right-side in all animals. This finding is in an agreement with earlier studies [10] (for FB tracer injection into the cervical, paracervical and middle part of uterine horns) which found that the number of perikarya was always lower in the left side ganglia. Another significant fact is that since the decrease mentioned above was always more significant on the right-side in comparison to both groups of animals, we can speculate that the right-side neurons of PCG must play a more important role in the innervation of this organ. The fact that uterine inflammation has an impact on the functioning of the reproductive system, like ovaries where it may cause pathologic folliculogenesis, cysts formation or elongate the luteal phase is well known [4, 28, 29]. Therefore, as mentioned in the authors’ previous articles on the current study [24, 25], the decrease in the numbers of uterine-perikarya may be treated as a consequence of both the endometritis and the decrease in the E2 level. This may also imply that an increase in the androstenedione level in pigs and cows results from it directly [29–31]. In the last decade, it was revealed that long-term E2 and testosterone administration decreases the number of ovary-innervating paracervical ganglion perikarya in the CaMG [32, 33], sympathetic chain ganglia (SChGs) [34], DRGs [35] and PCG as well [36, 37]. In the available literature, the estrogen role has been determined to be neuroprotective, as it protects neuronal perikarya from fatal damage. Such action is possible due to their ability to activate intracellular signaling pathways, as well as estrogen receptors (ERs) [38, 39]. Such receptors can be commonly found in DRGs neurons innervating the uterus [40], porcine ovary neurons in the CaMG [32], as well as in sympathetic and parasympathetic neurons in the PCG of rats and pigs [36, 41]. Moreover, a decrease in the population of ER-immunoreactive perikarya has been reported as well as a simultaneous drop in the ovarian neuron numbers in PCG in response to a long-term E2 treatment [36]. On the other hand, long-term testosterone supplementation of gilts leads to an increase in the androgen receptor-expressing ovarian perikarya with a simultaneous decrease in the total number of these type of neurons in the PCG [37]. The high probability of direct uterine inflammation impact on the seriously diminished numbers of uterine perikarya may be further supported by multiple reports of higher levels of pro-inflammatory cytokines in the course of uterine inflammation. These include substances such as TNF-α, IL-1β or PGF2α [4–6]. All mentioned cytokines are capable of the generation of free radicals while taking part in neuronal apoptosis [42]. This is why an alteration in uterine-perikarya in PCG can be connected to the above-mentioned facts, and could explain the similar values of estrogen and androgen receptors as well as inflammatory mediators receptors levels for the animals treated with bacterial inoculation. It is important to note that the decrease in PCGs neuronal numbers associated with steroid hormones and pro-inflammatory factors has not been sufficiently studied and requires more research.

Concerning changes in the chemical coding of uterine-perikarya in PCG, there were no statistically significant alterations in DβH-, VIP-, SOM-, nNOS- and NPY-immunoreactive neurons between the control and saline groups. This implies that either surgical actions and/or saline injections do not have any impact on the immunoreactivity of the examined perikarya.

The current study revealed a rise in the number of neuronal populations expressing DβH and VIP, perikarya positive for DβH and SOM, DβH and NPY, as well as DβH and nNOS. An increase was additionally found in neurons DβH-negative but VIP-positive, DβH-negative but SOM-positive and DβH-negative but NOS-immunoreactive populations. In contrast to that, drops in the number of DβH-immunopositive but VIP-, NPY- and nNOS-negative perikarya, as well as in neurons with no staining to any of the substances were presented. Similar to these results, in the authors’ earlier study, a rise in large uterine-supplying, CaMG-located perikarya positive for DβH and VIP, as well as DβH and NPY, was reported [25]. To the contrary, other authors described the number of DβH+/VIP+, DβH+/NPY + and DβH+/SOM + ovary-innervating neurons both in CaMG and SChG decreasing in response to prolonged 17β-estradiol treatment in sexually mature gilts [32, 34]. A decrease in TH, NPY and VIP expressing CaMG-neurons was also presented for chemically induced colitis in pigs [43]. It is worth noting that the testosterone treatment caused an increase in the number of noradrenergic DβH-expressing ovarian neurons in immature gilts [37]. What is more, a recent study on the effects of Bisphenol A, which has a known impact on estrogen receptors, especially ER-α, clearly shows that even small doses of this substance have a measurable impact on the number immunopositive nerve fibers located either in the right or left horn of the porcine uterine body. In this experiment, it is interesting that each uterine area noted a significant increase in populations of DβH and VIP-immunopositive fibers, as well as those positively stained for DβH and nNOS [44]. It is, of course, possible that some part of these are indeed perikaryal terminals of other sympathetic ganglia supplying the uterus, however, it is in line with the current study in which a population of DβH/nNOS-immunoreactive perikarya appears. As these studies are the first to reveal changes in the chemical coding in the PCG of mature gilts, this suggests that such nNOS/DβH-immunopositive neurons appear in animals after sexual maturation, whereas the rise in the E. coli-treated population may be a direct neuronal response to the uterine inflammation. Earlier studies revealed the existence of nNOS-immunoreactive perikarya in co-localization with VAChT [17]. However, a neuronal population immunoreactive to TH in co-localization with nNOS has been described in other studies on the immunohistochemical features of the PCG supplying the oviduct [45] although the animals used in that experiment were not sexually mature.

As mentioned earlier, a decrease in noradrenergic VIP-, SOM- and nNOS-negative perikarya was noted in the bacteria-treated group, which is testified by the results obtained in ovary-innervating neurons of SChGs and CaMG of sexually mature gilts treated with E2 [32, 34], as well as in CaMG uterine-supplying cells from the authors’ previous studies [25]. Additionally, a drop in the numbers of DβH negative and NPY positive perikarya similar to the drop found in the current study was also reported in one of the authors’ recent studies [25]. Moreover, the non-sympathetic populations expressing VIP, SOM and nNOS in the E. coli group increased in the current study. The mentioned alterations might indicate the disrupted variety of noradrenergic and non-sympathetic mechanisms. It has been proven that noradrenaline, which activates α- and/or β‐adrenergic receptors and enhances PGs production, inhibits the contractility of the uterus, either in its physiological or pathological state [46–49]. Similarly, VIP and NPY also play a role in contractility regulation [50, 51]. Moreover, VIP, best recognized due to its anti-inflammatory functions, is also known for its neuroprotective capabilities, which include enhancing neuronal survivability in cells under pathological conditions, such as inflammations [52–54]. NO is also known for its neuroprotective role in both central and enteric nervous systems under optimal conditions [18, 55, 56]. NPY controls the blood flow in vessels and is invaluable due to its neuroprotective properties [57–60]. SOM, on the other hand, is able to adjust motility and cell proliferation in the endometrium [61]. The elevated numbers of VIP, NPY, SOM and nNOS expressing perikarya, either noradrenergic or not, may indicate an increasing demand to upregulate these valuable neurotransmitters in direct response to metritis/endometritis, in order to benefit from their neuroprotective and anti-inflammatory properties. In addition, some of the above-mentioned substances, like NPY and SOM, might have a supportive role in the process of exudate removal in the inflamed uterus due to their contractility-enhancing capabilities. In the practical aspect, the collected data may contribute to the development of new methods of prevention and treatment of uterine inflammations. It might also lead to the improvement of reproductive indicators, as well as fewer animals eliminated from breeding and increased production profitability.

## Conclusions

In gilts, uterine inflammation due to E. coli resulted in alterations in numerical and neurochemical patterns of PCG uterine perikarya. Degradation of innervation might point to the fact that this organ’s immunity, adaptation capabilities and regulatory potential are all significantly altered. Moreover, various changes in the number of sympathetic or non-sympathetic uterine neurons expressing different neurotransmitters imply that the inflamed state of the examined organ may have a strong impact on such neurons. The obtained results further assure that the pig model is still well-suited for research on the pathological states strictly related to animal breeding, as well as human beings, including studies on the reproductive system. These results may confirm the sufficient impact of the inflammation on the chemical coding of the involved neurons. Moreover, the expanded knowledge may be utilized to develop new therapeutic analogues of neurotransmitters to help the uterus to return to its normal functioning.

## Methods

### Animals

The described research was performed on 11 sexually mature, crossbred gilts acquired from the “Wronie” breeding farm located in Wronie, Poland. Each being in the age of 7–8 months and weighing 90–120 kg. The animals showed signs of behavioral estrus, which was confirmed with the use of a tester boar. After unloading, all were randomly divided into 3 subgroups: A – E. coli group (*n* = 4), B – saline group (*n* = 3) and C – control group (*n* = 4), each located in different pens. The animals had three days for adaptation, after which the experiment began. Gilts subjected for the research did not have any disturbances in reproductive processes and were kept in normal laboratory conditions inside the animal quarters of the Faculty of Veterinary Medicine of the University of Warmia and Mazury in Olsztyn, Poland. Feeding was standard for this species and age and water was available *ad libitum.* All animals were kept in 5 m^2^ individual pens, with 14.5 ± 1.5 h of natural light during the day and 9.5 ± 1.5 at night. The temperature was kept at 18 ± 2 °C in accordance with the instructions and agreement of the Local Ethical Committee in Olsztyn, Poland. Stress reaction connected to surgery and the time after was minimized in accordance with Consent no. 65/2015.

### Experimental procedures

The procedures were as follows: on day 0 of the experiment (day 17 of the first studied estrous cycle) all animals received premedication consisting of atropine (0.05 mg/kg of body weight /BW/, injected intramuscularly; Atropinum sulf. WZF, Warszawskie Zakłady Farmaceutyczne Polfa S.A., Poland), azaperone (2 mg/kg BW, injected intramuscularly Stresnil, Janssen Pharmaceutica, Beerse, Belgium) and ketamine hydrochloride (10 mg/kg BW, injected intravenously; Ketamina, Biowet, Puławy, Poland). General anesthesia was induced with the use of ketamine hydrochloride (10 mg/kg BW, injected intravenously; Ketamina, Biowet, Puławy, Poland). It was then sustained with calculated doses of the same anesthetic (1 mg/kg BW applied after every five minutes, injected intravenously).

The subsequent abdominal incision was used to locate and expose the left and right uterine horns, after which a 5 % aqua solution of FB retrograde fluorescent neuronal tracer (EMS-CHEMIE, GmbH, Germany) was thoroughly injected using a 26-gauge needle of a Hamilton syringe into the wall of both horns. Its tracing capabilities made the bodies of the uterine supplying neurons visible. The tracer was applied into three parts of horns – paracervical, middle and paraoviductal – each treated with 13 separate FB injections (with a 2 cm injection ring diameter, single injection volume 2 µL and total − 26 µL). Due to FB leakage possibility the needle was left stationary for 60 s after each application. Injection areas were further rinsed with isotonic saline and wiped with gauze. The retrograde marker requires 4 weeks to reach the extrinsic sources of innervation.

With 28 days passed, all animals were anesthetized for the second time on the expected day 3 of the third studied estrous cycle using the same procedure as described earlier. Subsequent laparotomy allowed to inject either 50 mL of E. coli suspension (in case of the E. coli group; strain - O25:K23/a/:H1; 109-colony forming units/ml; National Veterinary Research Institute, Department of Microbiology, Puławy, Poland) or 50 mL of saline solution (for the saline group) into each uterine horn, whereas the control group received laparotomy only. On the expected 11th day of the third studied estrous cycle (8 days after laparotomies), all animals were euthanized by an overdose of an intravenously-injected ketamine hydrochloride. Then all animals were perfused with a 4 % buffered paraformaldehyde via the ascending aorta and subjected for PCG collection along with the whole uterine cervix, as well as the ligamentum latum uteri. Moreover, parts of bladders were collected together with cervices to enhance spatial orientation. After collection, the tissues were postfixed by immersion in a fixative for 10 min and then washed with 0.1 M PB (pH 7.4) over the course of 2 days. Finally, these were stored at 4 ºC in an 18 % buffered sucrose solution (pH 7.4) with an addition of 0.001 % natrium azide. Until further procedures, the ganglia were kept at -80 ^o^C. In order to determine the form of the inflammation, the fragments of uterine horns were fixed in a 4 % paraformaldehyde solution (pH 7.4) for 24 h, and the tissues were then washed in 0.1 M phosphate buffered-saline (PBS, pH 7.4) and embedded in paraffin. The findings of the uteri histological assessment were published previously [24].

### Immunohistochemical analysis

Collected uterine cervices were frozen together with PCG, cut in a cryostat (Frigocut, Reichert-Jung, Nussloch, Germany) into 14 μm thick sections and mounted on chrome alun-coated slides. Using an Olympus BX51 microscope (Olympus, Poland) with an epi-illumination fluorescent microscopy module (V1 module, excitation filter 330–385 nm, barrier filter 420 nm), the presence of FB-positive uterine neurons was checked in the examined sections. All sections with FB-labeled neurons were subjected to immunohistochemical procedures, which consisted of a routine double-labeling immunofluorescence technique. After air-drying at room temperature for 45 min. and rinsing in 0.1 M phosphate-buffered saline (PBS; pH 7.4) three times for the duration of 10 min. each, sections were incubated in a blocking buffer containing 10 % of normal goat serum in 0.1 M PBS, 0.1 % donkey serum, 1 % Triton X-100, 0.05 % thimerosal and 0.01 % NaN_3_ for 60 min. at room temperature in order to reduce non-specific staining background. Subsequently, after another wash in PBS (three times for 10 min.), the sections were incubated overnight at room temperature with primary antisera against the DβH and/or SOM, VIP, nNOS, and NPY.

The following day sections were rinsed with PBS 5 times for 15 min. and incubated with secondary antibodies (Alexa Fluor 488 and Alexa Fluor 546) suspended in PBS containing 0.25 % BSA and 0.1 % Trition X-100 for 1 h to show DβH/SOM, DβH/VIP, DβH/NPY and DβH/nNOS antibody combinations. The exact specifications of antibodies used are presented in Table 1. The sections were then rinsed with PBS three times for 5 min. and covered with a glycerin solution containing DABCO (Sigma, USA). Standard controls, i.e. pre-absorption for the neuropeptide antisera with appropriate antigen (20 µg of antigen/ml diluted antiserum) and the omission as well as the replacement of all primary antisera by nonimmune sera, were performed to test immunohistochemical labelling. There was no fluorescence observed in any of these control stainings.

**Table 1:** Antibodies used for immunostaining procedures

**Primary Antibodies**				
**Antigen**	**Code**	**Host Species**	**Dilution**	**Supplier**
DβH	AB1585	rabbit	1:500	Sigma-Aldrich, Saint Louis, MO, USA
VIP	ABS 023 − 02	mouse	1:1000	ThermoFisher Scientific Waltham, MA, USA
SOM	8330-0009	rat	1:60	Bio-Rad Laboratories, Watford, United Kingdom
NPY	ABS 028-08-02	mouse	1:1000	ThermoFisher Scientific Waltham, MA, USA
nNOS	N218	mouse	1:1000	Sigma-Aldrich, Saint Louis, MO, USA
**Secondary Antibodies**				
**Reagent**	**Code**		**Dilution**	**Supplier**
Alexa Fluor 488 nm donkey anti-mouse IgG	A21202		1:1000	ThermoFisher Scientific Waltham, MA, USA
Alexa Fluor 488 nm donkey anti-mouse IgG	A11010		1:1000	ThermoFisher Scientific Waltham, MA, USA

After staining, FB-labeled/double-immunostained neurons were further checked under a fluorescent microscope to count and analyze visible antibody combinations and then photographed with a digital monochromatic camera (Olympus XM 10) connected to a PC. All DβH-, SOM-,VIP-, nNOS- and NPY-immunoreactive and/or all retrograde-labeled cell bodies were counted in every fourth section of the PCG. All uterine perikarya, aside from their diameters, were accepted into one size class. Differences were considered significant at *p* < 0.05.

### Statistical analysis

Data gained from PCGs of control (*n* = 4), saline (*n* = 3)- and bacteria (*n* = 4) -treated gilts, was averaged per total number of ganglion perikarya, the population of nerve cells in left- and right-side ganglia and perikarya with particular chemical coding for each group. Data are expressed as percentages of the total population of uterine perikarya stained for two substances in each group, accepted as 100 %. To calculate the standard error of mean (± SEM), a one-way analysis of variance (ANOVA) followed by the Bonferroni test was used. All statistical analyses were performed using Statistica 13 software (StatSoft Inc., Tulsa, OK, United States). The differences were considered significant at *p* < 0.05.

## Data Availability

Not applicable.

## Abbreviations

PCG: Paracervical ganglion
DβH: Dopamine-β‐hydroxylase
NPY: Neuropeptide Y
SOM: Somatostatin
VIP: Vasoactive intestinal polypeptide
nNOS: Neuronal isoform of nitric oxide synthase
E. coli: *Escherichia coli*
IL: Interleukins
NO: Nitric oxide
PG: Prostaglandin
LT: Leukotriene
VAChT: Vesicular acetylcholine transporter
ChAT: Choline acetyltransferase
NA: Noradrenaline
TH: Tyrosine hydroxylase
NANC: Non-adrenergic, non-cholinergic
GAL: Galanin
SP: Substance P
DRGs: Dorsal root ganglia
CaMG: Caudal mesenteric ganglion
FB: Fast-Blue
PBS: Phosphate buffer solution
ER: Estrogen receptor
TNF: Tumor necrosis factor
