# Correction: Endometritis decreases the population of uterine neurons in the paracervical ganglion and changes the expression of sympathetic neurotransmitters in sexually mature gilts

**DOI:** 10.1186/s12917-022-03406-1

**Published:** 2022-09-10

**Authors:** Bartosz Miciński, Barbara Jana, Jarosław Całka

**Affiliations:** 1 Department of Clinical Physiology, Faculty of Veterinary Medicine, University of Warmia and Mazury, Oczapowskiego 13, 10-719 Olsztyn, Poland; 2 Division of Reproductive Biology, Institute of Animal Reproduction and Food Research, Polish Academy of Sciences, Tuwima 10, 10-748 Olsztyn, Poland

**Correction: BMC Vet Res 17, 240 (2021)**

**https://doi.org/10.1186/s12917-021-02949-z**

In our BMC Veterinary Research publication [1] entitled ‘Endometritis decreases the population of uterine neurons in the paracervical ganglion and changes the expression of sympathetic neurotransmitters in sexually mature gilts’, we incorrectly described the method of euthanasia. Ketamine (Vetaketam, Vet-Agro, 15 mg/kg i.m.) was given to the pigs as a premedication, which rendered them unconscious. Euthanasia was then performed through an IV overdose of sodium pentobarbital (Morbital 1ml/10kg). Death was confirmed by the determination of lack of pulse, breathing, corneal reflex as well as lack of response to toe pinching and the inability to hear heartbeat and respiratory sounds with the use of a stethoscope.
